# General population’s perceptions regarding marriage in patients with schizophrenia

**DOI:** 10.1192/j.eurpsy.2025.2277

**Published:** 2025-08-26

**Authors:** Y. Trabelsi, B. N. Saguem, N. Jaafar

**Affiliations:** 1 Psychiatry Department, Farhat Hached Hospital, sousse, Tunisia

## Abstract

**Introduction:**

Public perceptions of mental illness, particularly in the context of marriage, are shaped by a myriad of factors, including cultural beliefs, media representations, and personal experiences. These perceptions often involve misconceptions and negative stereotypes leading to social stigma and discrimination. For individuals with schizophrenia, stigma can result in significant challenges in forming and maintaining marital relationships.

**Objectives:**

To explore these perceptions we performed a qualitative analysis of public attitudes toward marriage in individuals with schizophrenia. By examining these perceptions, we aimed to identify common themes, misconceptions, and areas where public education might be most effective.

**Methods:**

A cross-sectional study was conducted among general population. A survey was proposed via social networks. It included, additionally to socio-demographic and clinical variables, a detailed description of clinical symptoms and outcomes of schizophrenia along with open-ended questions assessing the perceptions of potential benefits and disbenefits of being an intimate partner of an individual with schizophrenia. Answers were arranged and the commonly reported themes in public opinion were identified.

**Results:**

A total of 304 participants majoritively aged between 20 and 30 years old were included, 80.9% among them were women. The most of them were graduated from university. Family psychiatric history was reported by 35.6% of the participants and 23.35% of them stated that they were living with a person with a psychiatric disorder. About 87% of the participants admitted that they had seen a psychiatrist at least once in their lives. Responses regarding potential disbenefits of being married to a patient with schizophrenia included 8 themes, the most representative ones were 1) the fear of dealing with the exacerbations (16.4%); 2) the risk of being attacked physically (10.9); 3) the impossibility of maintaining a stable relationship (10.9%); 4) the lack of communication (6.6%); and 5) the risk of separation (5.6%). Other themes included concerns about 6) dealing with an irresponsible partner (4.6%); 7) the risk of suicide and 8) the possibility of transmitting the pathology to descendants (2.3%).

About 23% of the participants stated that they were willing to get married with a schizophrenic patient in order to help him survive his illness.

**Conclusions:**

This study has shed light on the complex and multifaceted perceptions held by the general population regarding marriage with a schizophrenic person. However, the study also revealed a glimmer of hope in the form of empathy and a growing recognition of the need for accurate information and support. As public awareness of mental health issues continues to rise, there is potential to challenge and change these negative perceptions.

**Disclosure of Interest:**

None Declared

